# Community-driven computational biology with Debian Linux

**DOI:** 10.1186/1471-2105-11-S12-S5

**Published:** 2010-12-21

**Authors:** Steffen Möller, Hajo Nils Krabbenhöft, Andreas Tille, David Paleino, Alan Williams, Katy Wolstencroft, Carole Goble, Richard Holland, Dominique Belhachemi, Charles Plessy

**Affiliations:** 1 University Clinics of Schleswig-Holstein, Department of Dermatology, formerly University of Lübeck, Institute for Neuro- andBioinformatics, Ratzeburger Allee 160, 23530 Lübeck. Germany; 2 Debian Linux; 3 Spratpix, Am Kiel-Kanal 2, 24106 Kiel, Germany; 4 Università degli Studi di Palermo, Dipartimento di Scienze Stomatologiche, Via del Vespro 129, 90127 Palermo, Italy; 5 University of Manchester, Oxford Road, Manchester,M13 9PL, UK; 6 Eagle Genomics, Babraham Research Campus, Cambridge CB22 3AT, UK; 7 Section of Biomedical Image Analysis, Department of Radiology, University of Pennsylvania, 3600 Market Street, Suite 360, Philadelphia, PA 19104, USA; 8 RIKEN Omics Science Center, 1-7-22 Suehiro-cho, Tsurumi-ku, Yokohama City, Kanagawa, 230-0045, Japan

## Abstract

**Background:**

The Open Source movement and its technologies are popular in the bioinformatics community because they provide freely available tools and resources for research. In order to feed the steady demand for updates on software and associated data, a service infrastructure is required for sharing and providing these tools to heterogeneous computing environments.

**Results:**

The Debian Med initiative provides ready and coherent software packages for medical informatics and bioinformatics. These packages can be used together in Taverna workflows via the UseCase plugin to manage execution on local or remote machines. If such packages are available in cloud computing environments, the underlying hardware and the analysis pipelines can be shared along with the software.

**Conclusions:**

Debian Med closes the gap between developers and users. It provides a simple method for offering new releases of software and data resources, thus provisioning a local infrastructure for computational biology. For geographically distributed teams it can ensure they are working on the same versions of tools, in the same conditions. This contributes to the world-wide networking of researchers.

## Background

The field of bioinformatics has gained momentum over the past two decades. The wealth and heterogeneity of biological data available in the public domain provides rich resources for bioinformaticians, biologists, chemists and clinicians. The latest Nucleic Acids Research databases special issue [1] recorded over 1000 biological data resources. This reflects the technological advancements in the field and the complexity of the field that has developed many sub-disciplines.

Users of biological data resources, especially in clinical environments and whenever biological processes are modelled, need an integration of those specialised resources. If we consider that many data resources have a collection of analysis tools associated with them, then the huge potential for combining these tools for analysis is traded off for the technical complexity. Many of these tools run on a command line, where there are differences in formats and the semantics of files to be exchanged between them. The installation and maintenance of these tools can introduce large overheads to data analysis. Alternative web-based interfaces are provided for many tools. This reduces the installation overhead for the user and the propensity for version heterogeneity, but it also often reduces the function of the tools. Users can submit large batch jobs, but they are limited to numbers or time.

Workflows provide the possibility of programmatic access to distributed tools and resources. The Taverna workflow workbench [2] allows users to chain together pre-made building blocks of web services and other services to build complex analysis pipelines. The myExperiment [3] site allows for the sharing of these workflows, helping people share informatics methods in the community. This way, researchers are helped to avoid reinventing approaches and prefer reusing established templates if readily available. This works nicely for public data and web services.

A challenge remains to integrate public services with the freedom to execute command line applications. The incorporation of command-line applications in such workflows is a more recent development, not yet widely adopted. We will explain why we think that this is not surprising and how the availability of Linux distributions contributes to a wider acceptance of that principle.

Applications may be executed either locally or remotely, possibly as a service, in a queuing system or a computational grid. The community has become excellent at sharing data, and similarly excellent in sharing the code (data) of applications, but there was yet no environment allowing for the integration of it all – either locally or remotely. The researcher demands the highest responsiveness from his applications and needs to protect precious data, especially in pharmaceutical or clinical environments. Both are in strong favour of local installations for applications and public data resources.

The traditional installation of Open Source software requires its compilation from source code. The skills and interest needed for packaging software are different from software development. More complex software will require the prior installation of other software, a recursive process. This is laborious and often technically challenging because of the differences between platforms and version incompatibilities. A workflow demanding the availability of a particular application on a local machine will thus be considered non-executable, unless the given application has already been installed.

Once installation is successful, scientists are reluctant to update a working environment, so computer networks quickly become heterogeneous and not maintainable with multiple users. When different members of a community work with different versions of the same software, it becomes more difficult to collaborate.

On Linux, several distributions are preparing packages with readily usable software that are up- and down-gradable, and allow libraries of several versions to be installed. Debian [4], founded in 1993, is one of the oldest distributions. It was for many years the only distribution that was managed as a democratic society, to which users could upload packages and vote for their destiny. The community of computational biologists should team up with the community behind the Linux distributions and extend the IT infrastructure respectively. This frees considerable resources for research labs and may be crucial for many smaller groups.

The remaining challenge was to connect those tools with the data and web services. One can certainly use various forms of direct access to download complete databases and experience a complete working environment. However, the prospect of integration with Taverna and therefore being able to access web services and workflows directly seemed particularly attractive. A Use-Case plugin was developed [5] to allow regular applications to be interpreted as services by Taverna and can be accessed in a similar way to web services.

The dependence of the biological research community on computational and data services will increase over the upcoming years. The strong computational demands of the services and the increase in complexity of the *in silico* research fosters the collaboration of individuals from many sites.

## Implementation

### Debian-Med

The Debian project is an open society of enthusiasts from around the globe who collaborate on maintaining an operating system based on the Linux and FreeBSD kernels. Programs are distributed as binary packages ready for use, built on Debian's network of autobuilding [6] machines, from source code that is further annotated and uploaded as packages by individuals. Debian supports today's most prominent platforms, thus rendering them available from mobiles to supercomputers and for all common processors. Packages invite feedback from users with the Bug Tracking System [7].

Around 90,000 users have allowed the counting of their installations via Debian's Popularity-Contest initiative [8], started in 2004. Separately counted are installations of packages that were forwarded to derived distributions [9]. The most prominent of these is Ubuntu [10], for which more than 1.3 million users are reporting. Packages are described verbosely and are translated to many languages [11]. More formally they may be selected by manual assignment of terms from a controlled vocabulary [12].

Technical constraints for the packaging are laid out in the Debian Policy document [13]. Changes to it are discussed on the project's mailing lists and may be subject to voting by contributors to the distribution. The Ubuntu Linux distribution adopts the Debian packages for their own software "universe" and several packages are co-maintained by developers of both distributions.

Everybody can volunteer to maintain a package in Debian. There is no general exclusion of any software, as long as it is redistributable. For auto-building on many platforms, the source code and the libraries that it needs must be available. To allow for improvements, one must be allowed to edit the code. All this is more formally specified in the Debian Free Software Guidelines (DFSG).

For complex suites, packagers have an option to share their effort with the community [14]. Such group maintenance was made possible with the advent of Debian Blends [15]. A blend is a thematic flavouring of the distribution, with its own respective portal, source code management and mailing lists. This helps keep the Debian community together and attracts new users. The Blend's infrastructure displays the available software together with bibliographic and registration information. Users can thus cite and register, i.e. help the upstream developers to perpetuate their projects by showing their impact.

### Integration of the command line in workflows

Packages may come with multiple executables. Even with only a single binary, the exact parameter setting would not necessarily be clear from any given context. The respective packages' manual pages list a series of the most common contexts and describe the inputs, outputs and command line arguments for these. One also sees the need to sequentially execute multiple binaries on a single command line to complete one piece of elementary work.

To facilitate automatic execution, we introduced a description of such shell-based workflow elements in a machine-readable equivalent of a man page. We refer to it as “UseCases” since there may be multiple ways a program is used with very different parameter sets, but there should be only a single description for a tool to achieve a particular purpose. A single Debian package can therefore provide the application(s) for multiple such Use Cases. We have created a web-based repository to browse through a database of such use cases and offer a form to add new ones. Internal use is also well supported by allowing the user to download and locally maintain the collection of custom workflow elements as an XML file.

To bring the shell-executed workflow elements together with web services, we have developed a plugin to Taverna. The plugin reads the UseCase descriptions from a URL, and then controls the execution of the described programs on the user's behalf. Every Use Case is therefore available to be embedded in Taverna's regular workflows and MIME types can be set to help with visualisation of final or intermediate results.

## Results and discussion

This section summarises the results of the Debian Med [16][17]* Blend* for medical- and bioinformatics. It is unique in its form, since all its packages are intrinsic parts of Debian. The Blend does not extend the distribution, it is simply a filtered view of what is available for the Life Sciences. Its packages help descendent efforts like BioLinux [18].

Debian Med provides a web portal interface, allowing users to browse packages of interest and select specific tasks from those packages. For bioinformatics, tasks of particular interest include “imaging”, “statistics”, and “bio” or “bio-dev”. Packages with an emphasis on computation have also been collected under the task “cloud”. Every such task is associated with a regular Debian metapackage, allowing the easy installation of a whole set of packages at one time.

### Focus on software for medicine and bioinformatics

At the time of writing, Debian Med offered 83 packages for bioinformatics in accordance to the Debian Free Software Guidelines' demands on Free software, another 13 do not fulfil this criterion. A further 18 packages are co-maintained and may be built locally, but have never been requested as an integral part of the distribution. These preliminary packages are made available to help the community to work with programs with a license that does not allow the redistribution of their source code, or for which the packaging is not yet completed. Another 29 packages are for developing new applications, like those for the Bio* [19] programming libraries.

Even though the focus is on the bioinformatics packages, to package particular software often means first packaging many more general libraries that are needed as a build- or runtime-dependency. Those additional packages are not listed with Debian Med but will become one of 60,000 other regular Debian packages.

The Popularity Contest (PopCon) infrastructures of Debian and Ubuntu presents lower bounds for the number of installations of any package in the distribution. Common tools like T-Coffee are reported as installed 222 times in Debian and 1150 times in Ubuntu. The R package qtl [20] was reported 542 times, outrunning even Gromacs [21] 322 (2324 in Ubuntu), EMBOSS [22] 250 (1665) and AutoDock [23] 216 (797). This information is updated on a weekly basis and is thus a valuable extension of the information from the download statistics at the original site for grant writers. For Debian, PopCon also offers a graphical development of those numbers over time – showing increasing absolute numbers. When normalised to the total number of users, most packages show a decrease between the years 2004 and 2008, which may reflect increasing success of Debian outside the academic world, followed by a stabilisation or a modest increase.

However, when comparing those numbers with the average size of scientific conferences, then the immediate outreach of the packaging seems equivalent. It becomes obvious that the user base of Ubuntu for the scientific packages is around 6 times larger than for Debian. The total number of reporting users is about 18 times larger and given the practical equivalence of the two distribution for bioinformatics research, Ubuntu with its more frequent release cycles has the latest versions integrated sooner with its releases. This may have contributed to the observation of Debian's lower numbers for the development packages (BioPerl [24] was reported 177 times installed in Debian's PopCon system and 1611 times with Ubuntu, BioJava [25] 33 times with Debian (281 with Ubuntu)) or latest expensive technologies like for next generation sequencing data analysis with maq [26] (35 times with Debian and 388 installs reported for Ubuntu) or velvet [27] (35 and 339 reports). This trend may change since Debian now introduced official rebuilds of latest-version packages against the stable distribution, termed "backports" [28] and will be interesting to observe.

The distributions' installation statistics help the communication with the software developers. It is the first almost immediate feedback that they get from the distribution. Some developers, like those of AutoDock and BALLView [29], follow the distribution's bug reports directly or otherwise contribute to the presentation of their software in the distribution. They may be formulating a description of their package or upload new versions of their software directly to the distributions.

### Integration of tools and data

With an increasing number of packages available, the interaction between those tools becomes more important for analysis. At first sight, this addresses the establishment of regular workflows in bioinformatics that are expected to compose analysis pipelines from tools from many packages. A second issue is the challenge to extend the concept of packaging to the distribution of the exact same version of public databases on different sites. This is only an issue if the public instance cannot be used via the web directly, e.g. to avoid the risk of someone monitoring the query or because of a higher latency. The data are likely to update more frequently than the tools interacting with them, and the tolerance towards working solely with official releases varies between sites. The sharing of input between multiple applications is ongoing work, for which many bioinformatics groups around the globe have provided solutions independently. To tap into that wealth of experiences and use it to share the effort to maintain the infrastructure is our impetus.

Debian Med is currently investigating the acceptance of a Perl script, named getData [30], that knows how to install some of the most common databases in bioinformatics and how to do the post-processing to have that data readily available for EMBOSS, BLAST [31] or other associated tools. The information is stored in a hash table, and getData knows how to extend that hash from files in a configuration folder. This is a preparation for database packages that would only consist of such tiny configuration files that depend on getData and recommend a series of tools that know how to read the data. By some automatic mechanism in that package's post-install script or by a manual trigger by the user, the database would then be downloaded and indexed with no further human intervention.

However, there are limits in disk space and, more importantly, in the bandwidth available. Now that we are storing data of complete genomes for comparison, a shared environment should be identified, potentially in the cloud.

### Cloud computing for sharing a virtual computer

Cloud computing provides scalable, flexible access to larger computer processing power and storage. For academics, there are free cloud resources available (such as the National Grid Service Cloud in the UK [32]). Commercially, resources such as Amazon provide an on-demand service.

In clouds, virtual machines are used to instantly reconstitute one particular environment from a selection of images offered, or self-assembled, on one or more computers. This brings the advantage of letting scientists work on copies of the same system and further increases the reproducibility of workflows composed of local Debian Med services

Cloud computing offers an increased flexibility for workflow infrastructure in many ways. Rather than providing the service directly, a community may decide to offer a cloud image of the service. The images can be adapted for local needs or be run redundantly to avoid single points of failure. Multiple cloud instances can also be organised to be accessed together and share the work, e.g. in a manner known from high performance computing by installing a batch system [33].

In Debian Med, all packages are versioned and have set dependencies. This allows users to exactly specify a production working environment, which assists in the sharing of Debian Med images as well as in the collection of accurate provenance information. It is equally straightforward to adapt a public specification for local needs or to publicly discuss or automate the setup. Tools like live-builder.debian.net can create system images that are usable either on local computers or on large cloud services [34]. In addition, packages of arbitrary previous versions [35] can be substituted from the default ones, to assemble precise combinations of tools and thus generate system images that reproduce environments used for past projects.

The Amazon Public Data initiative [36] already exemplifies how bioinformatics databases of public interest can be shared in a cloud environment. Debian images can access and work with such data. And the community can, by a manual effort or by using getData, perform a similar initiative, albeit on a smaller scale.

### Workflows

The UseCase plugin provides a new service type in the Taverna Workbench. UseCase services present a mechanism for incorporating arbitrary command line tools into workflows. The command-line tools can be configured to run locally, or on remote machines accessed via secure credentials such as ssh or grid certificates. Multiple invocations of a service can be achieved by calling the same tool on a number of computing nodes at the same time, thus allowing faster running of workflows over a distributed network of machines.

Workflow developers can therefore write and test workflows locally on small amounts of data. A simple change of configuration can then run the workflow on a grid or cloud on much larger data sets.

**Figure 1:**
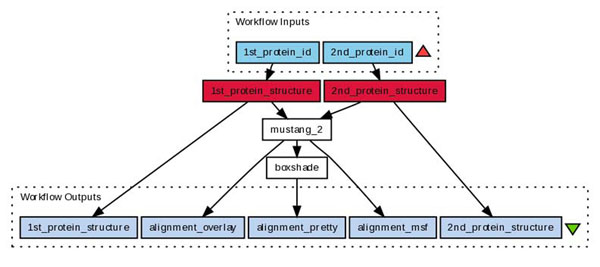
This graphical representation of a workflow in Taverna was taken directly from workflow 377 at the myExperiment site. It requests two PDB IDs to retrieve the respective protein structure and present it to the mustang protein structure alignment tool. Its result files are directly offered also as outputs of the workflow. It is only one alignment that is passed throught the tool boxshade for pretty-printing.

Taverna workflows can include not only tools within a packaged distribution, but also calls to other services such as WSDL operations, queries of a BioMart database or invocations of R scripts. The workflows can be uploaded to the myExperiment website to be shared, either publicly or with specific groups of people. Figure 1 presents an example workflow for the structural alignment program Mustang [37]. with the tool boxshade [38] to pretty-print sequence alignments. The original manuscript on the Taverna plugin [5] explains the syntax to describe the binaries in Debian or other Linux distribution to appear as workflow elements.

The development of workflows goes hand in hand with the sharing of such expertise via the myExperiment website. With the UseCase plugin, workflows could be composed entirely of packages from Debian Med. This renders the workflows more accessible for commercial in-house adoption, where data or methods are sensitive.

## Conclusions

The dynamics of the elements presented here, i.e. the Debian Med distribution, the cloud infrastructure and the workflow suite, form a symbiosis towards a readily usable infrastructure for performing and sharing biological research and services. Any command line application can be integrated with any workflow in Taverna via local or remote execution. Sharing workflows, services and data sources is not trivial, but can be managed successfully with the infrastructure presented here.

Cloud computing infrastructures offer a new way of working. Expensive local computing infrastructures are not required if researchers can have access to cloud computing resources on demand. Small research groups can therefore tap into resources that were previously not accessible to them [39].

## Authors' contributions

SM and AW drafted the manuscript, KW, CP, HK and SM revised it. HK implemented the use case interface to Taverna that was now adopted by AW. SM implemented the web interface to use cases and with CP prepared the getData script. AT, CP, DP and SM are all frequent contributors to the packaging for the Debian Med initiative.

## Availability and requirements

• **Project name:** Debian Med

• **Project home page:**http://debian-med.alioth.debian.org

• **Operating systems:** Debian Linux and derivatives

• **Programming language:** no restrictions

• **License:** any that allow the free redistribution of the software

• **Any restrictions to use by non-academics:** no (with exceptions for individual packages), none added by Debian

## List of abbreviations

AWS: Amazon Web Services; BLAST: Basic Local Alignment Search Tool; DFSG: Debian Free Software Guidelines; EMBOSS: European Molecular Biology Open Software Suite; MIME: Multipurpose Internet Mail Extensions; PopCon: Popularity Contest; URL: Uniform Resource Locator; WSDL: Web Service Definition Language;  XML: Extensible Markup Language;

## Competing interests

The authors declare that they have no competing interests.
